# Integrated structural and functional analysis of the protective effects of kinetin against oxidative stress in mammalian cellular systems

**DOI:** 10.1038/s41598-020-70253-1

**Published:** 2020-08-07

**Authors:** Muhammad Naseem, Eman M. Othman, Moustafa Fathy, Jibran Iqbal, Fares M. Howari, Fatima A. AlRemeithi, Geema Kodandaraman, Helga Stopper, Elena Bencurova, Dimitrios Vlachakis, Thomas Dandekar

**Affiliations:** 1 Department of Life and Environmental Sciences, College of Natural and Health Sciences, Zayed University, Abu Dhabi, UAE; 2 Department of Bioinformatics, Biocenter, University of Würzburg, Am Hubland, Wuerzburg, Germany; 3 Department of Biochemistry, Faculty of Pharmacy, University of Minia, Minia, Egypt; 4 Department of Regenerative Medicine, Graduate School of Medicine and Pharmaceutical Sciences, University of Toyama, Toyama, Japan; 5 Institute of Pharmacology and Toxicology, University of Würzburg, Würzburg, Germany; 6 Genetics Laboratory, Department of Biotechnology, Agricultural University of Athens, 75 Iera Odos str, 11855 Athens, Greece

**Keywords:** Biochemistry, Cell biology, Drug discovery

## Abstract

Metabolism and signaling of cytokinins was first established in plants, followed by cytokinin discoveries in all kingdoms of life. However, understanding of their role in mammalian cells is still scarce. Kinetin is a cytokinin that mitigates the effects of oxidative stress in mammalian cells. The effective concentrations of exogenously applied kinetin in invoking various cellular responses are not well standardized. Likewise, the metabolism of kinetin and its cellular targets within the mammalian cells are still not well studied. Applying vitality tests as well as comet assays under normal and hyper-oxidative states, our analysis suggests that kinetin concentrations of 500 nM and above cause cytotoxicity as well as genotoxicity in various cell types. However, concentrations below 100 nM do not cause any toxicity, rather in this range kinetin counteracts oxidative burst and cytotoxicity. We focus here on these effects. To get insights into the cellular targets of kinetin mediating these pro-survival functions and protective effects we applied structural and computational approaches on two previously testified targets for these effects. Our analysis deciphers vital residues in adenine phosphoribosyltransferase (APRT) and adenosine receptor (A2A-R) that facilitate the binding of kinetin to these two important human cellular proteins. We finally discuss how the therapeutic potential of kinetin against oxidative stress helps in various pathophysiological conditions.

## Introduction

The small-molecule adenosine N^6^-furfuryladenine (N6FFA: kinetin) is commonly used by the plant community as a low-priced proxy for the natural cytokinins (CKs) in plant tissue-culture experiments^1^. CKs are a group of phytohormones influencing the entire *bauplan* of plants; ranging from seed germination, cell division, flowering, organogenesis, immunity, and communication until senescence of the plant^1,2^. In plants, kinetin binds to almost all known CKs canonical pathway receptors and invokes analogous physiological responses as many more specific CK-types^2^. The naturally occurring CKs in plants are isoprenoid-type CKs, for instance, isopentenyl adenine (iP), *trans*-zeatin (*t*Z), *cis*-zeatin (*c*Z), and dihydrozeatin (DZ) are the most common forms^3^. The majority of naturally occurring CKs exist as free (active forms) bases. Cytokinins conjugate with sugars or amino acid residues and thus form inactive forms^4,5^. Previously, CKs were assumed to be exclusively present in the kingdom Plantae; however, their discovery in all forms of life except Archaea, have changed the former notion^6^. Likewise, land plants are considered to be the only eukaryotes that harbor two-components system (TCS) that senses and transduces the signal of CKs^7^. No such CKs-sensing circuitry has ever been reported for mammalian cells. More intriguingly, many human pathogens such as *Mycobacterium tuberculosis*^8^ and rodent malarial parasites such as apicomplexan parasites *Toxoplasma gondii* and *Plasmodium berghei* also produce and sense the presence of CKs^9^. These and alike studies underscore the biological significance of CKs in *ex-planta* cellular systems.

Besides their emerging biological implications for animal-host pathogen interaction systems, CKs have been gaining attention for their potential therapeutic roles as anticancer agents in vitro cell cultures^10,11^. Much emphasis has been placed on assessing new CKs with cytotoxic effects on cancer cell lines^12^. On the contrary, CKs has been used to mediate anti-ageing effects for fibroblast cells^13^, as a novel neuroprotectant^14^ and anti-inflammatory^15^ agent. Insights into the roles of kinetin through exogenous application in mammalian cells has gained attention through these studies; however, much remains to be explored about the in vivo metabolism and the endogenous function of CKs in mammalian cells. In this regard, Seegobin et al.^16^ unequivocally detected the presence of seven CK forms in a wide selection of canine tissues: iPRP (mono-, di-, and triphosphate), *cis*-zeatin riboside (*c*ZR), *cis*-zeatin nucleotide (*c*ZRP; mono, di-, and triphosphate), 2-methylthio-isopentenyladenine (2MeSiP), 2-methylthio-isopentenyl-adenosine (2MeSiPR) and 2-methylthio-zeatin riboside (2MeSZR) by mass spectrometry. Another interesting development is the metabolism of benzylaminopurine (BAP) in HeLa cells; a 12-fold decrease in BAPR concentration was observed within the first 24-h of incubation^17^ of BAP with HeLa cells. These findings point to the notion that mammalian cells possess the enzymatic pathways for the metabolism of both endogenous and exogenous CKs.

Kinetin was first isolated from human urine decades ago. However, its exact biogenesis in mammalian cells, its functional implications and toxicity effects were not intensively assessed until now. Recently, it was demonstrated that under sub-optimal availability of ATP, mammalian neuronal cells generate kinetin through Fenton reaction as a natural product of oxidative-DNA-damage^18^. Kinetin protects Huntington’s disease models in a dose dependent manner^18^, and that exogenous application of 1–10 µM concentration of kinetin improves the viability of the mutant huntingtin expressing cells with no significant toxicity effects on the neuron^18^. On the contrary, 1 µM exogenously applied kinetin was previously shown to increase cytotoxicity as well as genotoxicity in HeLa cells^19^. Quite recently, kinetin was shown to increase the inclusion levels of exon 20 of IκB kinase complex-associated protein (*IKBKAP)* in cells derived from familial dysautonomia (FD) patients; however, the effective dosage (10 µM) of kinetin in FD patients led to severe toxicity effects^20^. These various reports demonstrate opposing effects manifested by various doses of kinetin in diverse cell types under various pathophysiological conditions. Here, we systematically assessed the effect of kinetin concentrations on four different cell lines under conditions of oxidative stress.

Despite these emerging reports on the in vivo effects of CKs, it is of pivotal importance to identify and characterize CK/kinetin-binding protein (C/KBPs) within the mammalian cells. Previously, various soluble C/KBPs were found in mammalian sera^21^. Besides soluble proteins that bind CKs, 6-benzyladenine was also shown to interact with purinergic type-2 receptor, which is a membrane-bound protein that interacts with this type of CK in animal cells^22^. The anticancer effects of CKs were attributed to cyclin-dependent kinases^23,24^, the expression of their homologues in plants are regulated by CKs. Moreover, kinetin has been shown to restore N17 phosphorylation after being salvaged to its triphosphate type by the mammalian enzyme adenine phosphoribosyltransferase (APRT)^18^. Thus, kinetin seems to be a direct substrate of the APRT enzyme. Likewise, kinetin has been shown to act on the mammalian adenosine A2A receptor (A2A-R), and can be used as neuroprotectant^14^. These findings point to multiple-target sites in mammalian cells where CKs or kinetin can potentially bind. Elucidation of these various K/CBPs can be harnessed in unleashing the pharmaceutical potential of kinetin in various diseases.

In this study, we use four distinct cell lines to assess toxicity and protection mediated by different doses of kinetin. Moreover, we apply computational and structural biology tools to predict possible kinetin binding sites on APRT enzyme and A2A-R receptor. We discuss the significance of dose dependent kinetin responses in terms of C/KPBs and future directions in harnessing the pharmacological benefits of kinetin.

## Materials and methods

### Cell lines

All examined cell lines were obtained from the American Type Culture Collection.

HL-60, a human promyelocytic cell line, HL-60 cells were cultured three times per week at 37 °C, 5% (v/v) CO_2_ in RPMI 1,640 medium, supplemented with 10% (v/v) fetal bovine serum (FBS), 1% (w/v) l-glutamine and 0.4% (w/v) antibiotics (50 U/mL penicillin, and 50 mg/mL streptomycin).

HDF (Human Dermal Fibroblast), A549 cells, the adeno-carcinomic human alveolar basal epithelial cells and WI38, the a diploid human cell line composed of fibroblasts derived from lung tissue were cultured two times per week at 37 °C, 5% (v/v) CO_2_ in RPMI 1,640 medium, supplemented with 15% (v/v) fetal bovine serum (FBS), 1% (w/v) l-glutamine and 1% (w/v) antibiotics (50 U/mL penicillin, and 50 mg/mL streptomycin).

### Vitality test

Vitality staining was applied for the cell lines which were treated with different concentrations of kinetin for 24 h. 0.35 × 10^6^ cells were cultured in six well plates for 24 h in a control medium. After treatment with kinetin or H_2_O_2_ as positive control, cells were collected, and 2:1 mixture of the cell suspension and staining solution (Gel Red Biotrend, Germany; fluorescein diacetate) was prepared. 20 μL of this mixture was applied to the slide, 200 cells from each sample were examined at a 500-fold magnification with a fluorescence microscope for the fraction of green (viable cells) vs red (dead) cells.

### Comet assay

The alkaline version of the comet assay was applied^25^ in order to detect DNA single and double strand breaks in the kinetin treated cells. The method was applied to quantify the Kinetin-induced DNA damage in the mammalian cell lines. The following procedure were applied: (1 × 10^6^) cells were treated for 24 h with different concentrations of kinetin either alone or in combination with H_2_O_2,_ for 30 min post kinetin treatment.

The cells with all different treatments were harvested, a mixture of 20 µL of this suspension as well as 180 µL of 0.5% low melting point agarose were prepared. Cells were fixed on slides which covered with high melting point agarose (1.5%). To lyse the cell membrane and nuclear membrane, the slides were subsequently incubated for 1 h at 4 °C in lysis buffer (2.5 M NaCl, 0.1 M EDTA, 0.01MTris, and 10 g/L sodium *N*-lauroylsarcosine adjusted to pH 10) mixed with 1%Triton X-100 and 10% dimethylsulfoxide (DMSO). After washing the slides, they were placed for 20 min in electrophoresis solution which is a mixture of 5 M NaOH and 0.2 M EDTA and adjusted to pH 13.0. Electrophoresis was conducted for 20 min at 25 V and 300 mA, adjusted with the electrophoresis solution. Neutralization of the slides with 0.4 M Tris buffer (pH 7.5), was followed by fixation in cold methanol for 5 min at 20 °C. These methanol fixed slides were then dried at 37 °C for 10 min and afterwards stored at room temperature. Before evaluation, 20 µL of GelRed (1:100)/diazabicyclo-octane (1:4) solution was added to each slide and images of 100 cells at the middle of the slide (50 per replicate slide) were analyzed with a fluorescence microscope (Labophot2; Nikon GmbH) at 200-fold magnification using image analysis software (Komet 5; BFI Optilas). In three independent experiments we quantified (averaged) of the percentage of DNA in the tail as read-out of the DNA damage.

### Molecular docking and molecular dynamic simulation of kinetin to APRT enzyme and to A_2A_-R receptor

The molecular modelling of the Kinetin-APRT complex was performed using the Molecular Operating Environment Suite (MOE by CCG)^26^. The 1ORE, 3T4S and 2YDO RCSB entries were used for this experiment, which are the crystal structures of the human APRT protein, the AHK4 and A2A-R structures, respectively^25^. The 3D models were subsequently energetically optimized the CHARMM27 forcefield as it is implemented in the Gromacs Suite. The docking module of MOE was used for establishing the in-silico biological complexes of the Kinetin to APRT and A2A-R. A FFT—fast Fourier transformation pipeline is utilized by MOE for the docking experiment. The overall score is influenced by the model’s packing, electrostatic, solvation and hydrophobic energies. Transient complexes of proteins are kept in a local database and their contact propensities are statistically used for docking. The top hits of the docking experiment were energetically optimized using energy minimization pipelines to relieve the models from any residual geometrical strain. Finally, the Drugster suite was used to perform a final and rapid energy minimization step using an implicit Generalized Born (GB) water model. The interaction pattern and overall fold of the final three kinetin complexes was subjected to molecular dynamics simulations. Molecular dynamics simulations^27^ were executed in an explicitly SPC water solvated periodic cube system. Counter-ions were used as required to neutralize the molecular system. The two water solvated in silico kinetin complexes (APRT-kinetin and A2A-R-kinetin, with 34,385 and 99,981 atoms respectively) were subjected to one hundred nanoseconds of molecular dynamics at 300 K and at 2 fs step size^28^.

## Results and discussion

### Critical kinetin concentrations mediate cell viability and invoke cytotoxic responses from mammalian cells

In a previous study, we showed that in low concentrations (below 100 nM) kinetin protects mammalian cells against oxidative stress, whereas high concentrations (above 100 nM) exhibited an opposite activity as it induced genotoxicity and cytotoxicity in the treated cells^19^. We tested cells with diverse potencies and functional capabilities such as HL-60 cells, HaCaT human keratinocyte cells, NRK rat epithelial kidney cells and human peripheral lymphocytes. In all these cellular systems kinetin concentrations above 100 nM induced cytotoxicity in the treated cells^19^. The concentrations of kinetin that protect cells from cyto/geno-toxicity in other studies are far higher (µM-concentrations^13,18^) than those we used to rescue various cells from oxidative stress. To address this discrepancy, we redesigned experiments to find critical kinetin concentrations that cause cellular protection or mediate cytotoxicity to better ascribe kinetin functions in an era where it already got much attention for its therapeutic potential in mammalian cells.

**Figure 1:**
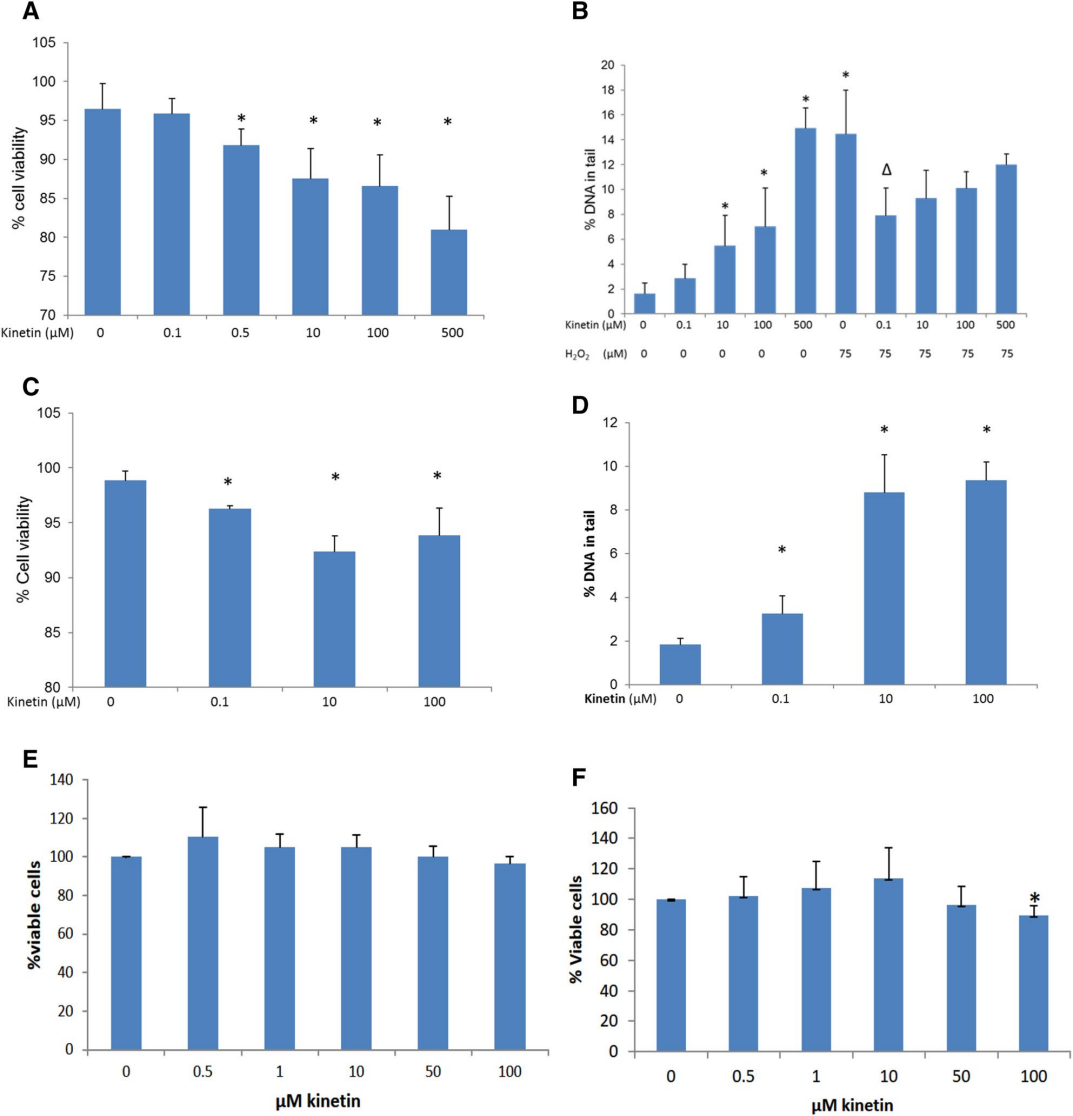
Survival and toxicity mediated by kinetin within mammalian cellular systems. (**A**) Vitality assay for HL-60; (**B**) comet assay for HL-60; (**C**) vitality assay for HDF; (**D**) comet assay for HDF; (**E**) vitality assay for A549; (**F**) vitality assay for WI38. Cells treated with different concentrations of kinetin and/or 75 µM H_2_O_2_ *Significantly different from negative control and ^∆^significantly different from H_2_O_2_ treated cells. Data are shown as averages ± SD from three independent experiments. Statistical significance among multiple groups was tested by the Kruskal–Wallis test. Individual groups were then tested using the Mann Whitney U-test and results were considered significant if the *p*-value was < 0.05.

We examined human promyelocytic HL-60 cells for cytotoxicity and genotoxicity after a 24 h treatment with low and high concentrations of kinetin. Cells which were treated with less than 500 nM kinetin did not show significant reduction in cell viability and no genotoxicity was induced. However, increasing the concentration beyond 500 nM we noticed cytotoxic as well as genotoxic effects of kinetin (Fig. 1A,B). We also exposed cells to 75 µM H_2_O_2_ in order to emulate conditions where cell burst occurs. A low dose of kinetin (0.1 µM) protected the cells against H_2_O_2_-induced genotoxicity, but by increasing the concentration of kinetin the protective effect was lost (Fig. 1B). It is noteworthy to mention that we did not see an additive effect in terms of genotoxicity between higher kinetin concentrations and addition of H_2_O_2_ to the cells. To confirm our results another cell line from different origin and with different potencies was examined. The human dermal fibroblast (HDF) cells were treated with low as well as high concentrations of kinetin. We got a similar trend in response to kinetin treatment of HDF cells (Fig. 1C,D) in comparison to the genotoxic and cytotoxic responses that are shown by HL-60 cells (Fig. 1A,B). The only difference in terms of kinetin treatment between these two cell types is the extent of the effect; the former seems relatively more sensitive than the latter. Furthermore, we treated WI38 cells with a range of kinetin doses (0–100 µM), we did not find a significant difference between mock treated and below 500 nM kinetin treated cells (Fig. 1E). However, a kinetin concentration of 500 µM significantly reduced cell viability as compare to mock treatment as well as lower (0–100 µM) concentrations. Likewise, the treatment of A549 cells with kinetin concentrations up to 100 µM did not show any significant reduction in cell viability (Fig. 1F).

We are of the view that kinetin mediates both protection as well as vulnerability of mammalian cells at lower and higher doses, respectively. In either case, the kinetin actions have interesting biomedical implications. The lower concentrations can be effective in pathophysiological conditions that lead to oxidative stress while higher doses may counteract cell proliferation by promoting apoptosis. We speculate that in higher and lower concentrations kinetin might have different cellular targets in invoking different cellular responses. Both these hypotheses pertaining cellular protection or cytotoxicity need detailed investigation involving small-molecule protein interaction studies as well as the metabolic fate of CKs in mammalian cells.

The focus of this work is to further analyze the pro-cell survival and longevity-like responses mediated by kinetin in conferring protection against oxidative stress in mammalian cells. On the other hand, the pro-apoptotic and cytotoxic phenotypes that kinetin causes in higher concentrations require an independent focus and study not followed further here.

To further substantiate the pro-survival functions of kinetin in favor of mitigating oxidative stress in structural detail we selected two previously testified targets of kinetin in mammalian cells for detailed structural biology analysis.

### Structural insights on human APRT metabolic enzyme as kinetin target in mammalian cells

**Figure 2:**
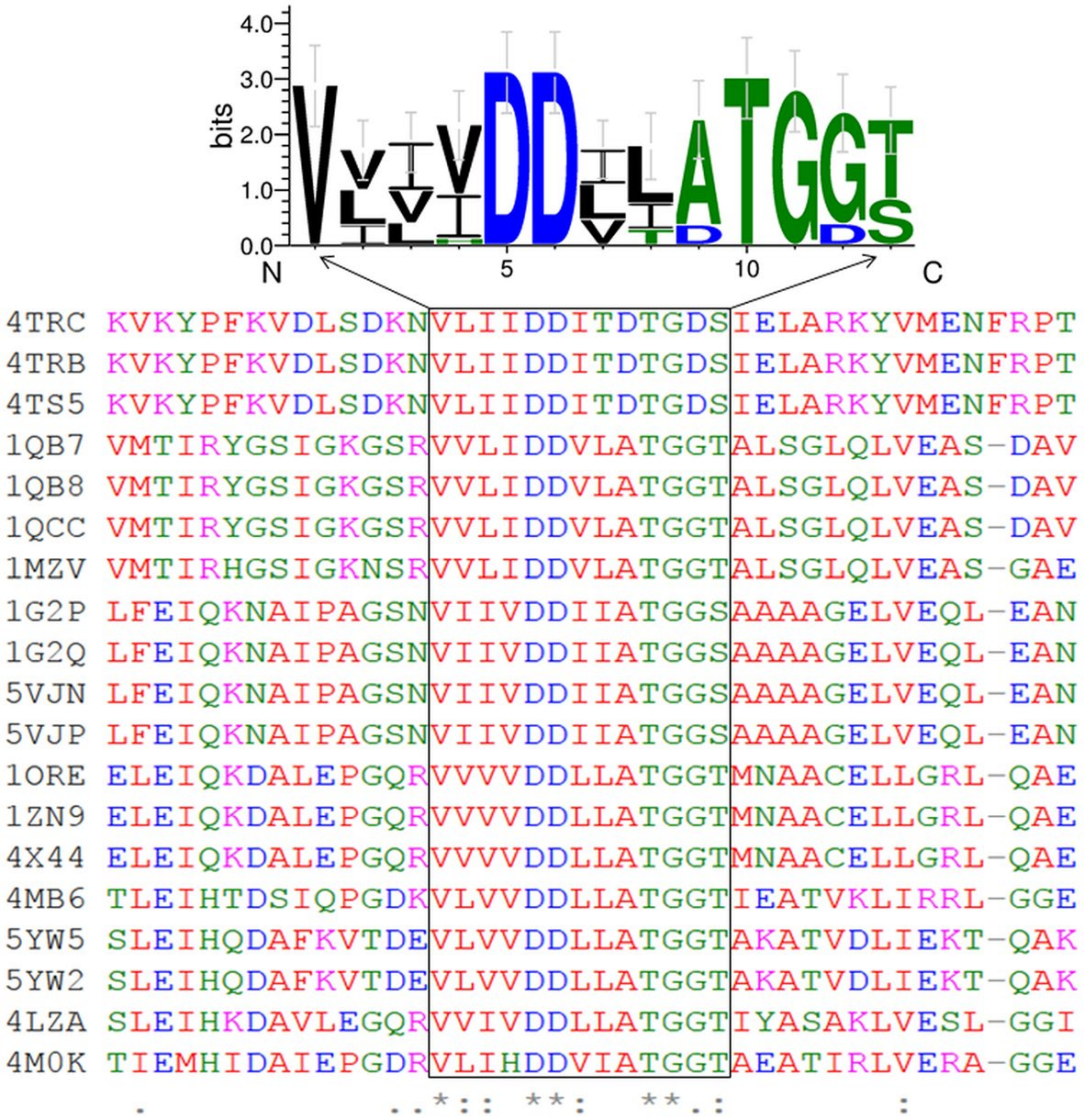
The conserved phosphoribosyltransferase (APRT) domain in various life forms. The multiple sequence alignment shows conservation among APRT enzyme sequences from various lifeforms: human, bacterial species e.g. *Francisella* sp., yeast (*Saccharomyces* sp.), protozoa (*Leishmania* sp.) and archaea (*Sulfolobus*). The catalytic signature is shown as logo on the top of the alignment.

**Figure 3:**
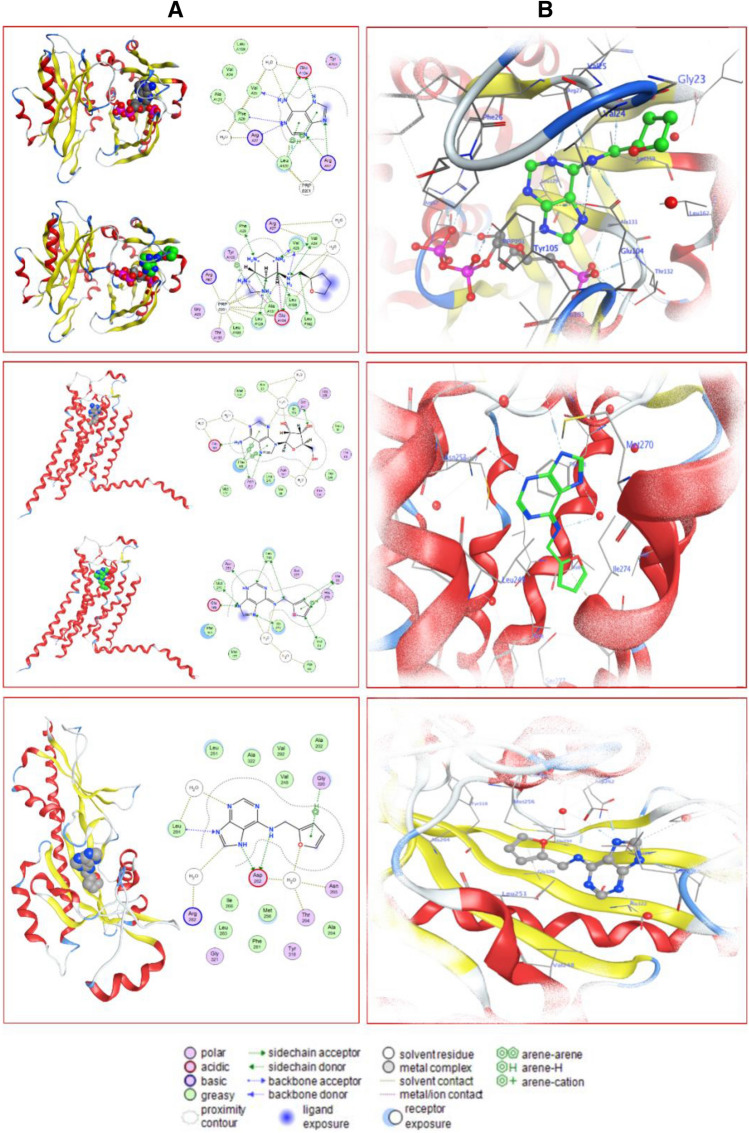
Kinetin binding interactions to APRT, A2A-R and AHK4. Upper panel: Kinetin and APRT (docking) (**A**): top is the molecular interactions of the adenine molecule and bottom with kinetin in the human APRT enzyme. The molecular interactions are showing for each molecule in 2D interaction maps. (**B**) The final pose of kinetin in the human APRT enzyme, while showing the amino acids it interacts with. Middle panel: Kinetin and A2A-R (docking) (**A**) top is the molecular interactions of the adenosine molecule and bottom with kinetin in A2A-R. The molecular interactions are showing for each molecule in 2D interaction maps. (**B**) The final pose of kinetin in A2A-R, while showing the amino acids it interacts with. Lower panel: Kinetin and AHK4 (crystal structure) (**A**): The molecular interactions of the Kinetin as crystalized in AHK4. (**B**) The crystalized pose of kinetin in AHK4, while showing the amino acids it interacts with.

It was recently demonstrated that mammalian cells harbor the enzymatic pathways for the metabolism of endogenously occurring and exogenously supplied cytokinin to cells^18^. The enzyme APRT, which is known to convert free bases in plants directly into the nucleotide fraction, has never been linked to a mammalian cellular system until recently^18^. To get further insights into the evolutionary conservation of APRT, we aligned 19 different protein sequences involving human, bacterial species (e.g. *Francisella* sp.), yeast (*Saccharomyces* sp.), protozoa (*Leishmania* sp.) and archaea (*Sulfolobus*) as representatives of various life forms of this important enzyme. We specifically focused on the catalytic cleft (Prosite signature PS00103) and found conserved residues across various taxa of life (Fig. 2). We, therefore, inferred that although previously considered as a synthetic CKs type, the kinetin can be synthesized by many different organisms belong to various life kingdoms. To study in more detail the ligand (kinetin) binding dynamics of the human APRT enzyme, we docked adenine (positive control) and then kinetin to the catalytic cleft of the enzyme (Table 1, Fig. 3). We visualized the molecular interactions of adenine (positive control) that is co-crystalized with the human APRT enzyme (Fig. 3 upper panel). We then replaced adenine with kinetin and found that the latter has occupied the conformational space of adenine and has established similar interactions with the enzyme. As indicated, many of the amino acids are conserved in the two docking interactions (Fig. 3 upper panel). The residues Val24, Val25, Phe26, Glu104, Tyr105, Leu129, Leu159 and Leu162 were found to interact in an adenine-like pattern using a repertoire of molecular interactions (Fig. 3 and Table 1).

**Table 1:** Enzyme–substrate binding report for adenine and kinetin binding to the APRT.

Ligand	Receptor	Interaction	Distance	E (kcal/mol)
**Adenine interactions to human APRT**				
N7 3	OE1 GLU 104 (B)	H-donor	2.69	− 6.3
N6 6	O VAL 25 (B)	H-donor	3.12	− 1.9
N6 6	OE1 GLU 104 (B)	H-donor	2.86	− 5.1
N9 1	NH2 ARG 67 (B)	H-acceptor	3.77	− 0.8
N6 6	O HOH 332 (B)	H-acceptor	3.61	− 1.2
N1 7	CA PHE 26 (B)	H-acceptor	3.43	− 0.5
N1 7	N ARG 27 (B)	H-acceptor	2.98	− 4.4
N3 9	NH1 ARG 67 (B)	H-acceptor	3.02	− 7.6
N3 9	NH2 ARG 67 (B)	H-acceptor	3.52	− 1.2
6-ring	CD1 LEU 129 (B)	pi-H	3.69	− 0.5
5-ring	CD2 LEU 129 (B)	pi-H	3.94	− 1.2
**Kinetin interactions to human APRT**				
C 1	CD1 LEU 159 (B)	H-donor	3.52	− 0.7
C 3	CD2 LEU 129 (B)	H-donor	3.04	− 0.6
C 5	OE2 GLU 104 (B)	H-donor	3.04	− 2.1
N 7	CE2 PHE 26 (B)	H-donor	3.71	− 0.5
N 13	CG1 VAL 24 (B)	H-donor	3.42	− 0.9
N 13	O VAL 25 (B)	H-donor	2.85	− 11.7
N 16	O4 PRP 201 (B)	H-donor	2.85	− 7.8
C 19	O5 PRP 201 (B)	H-donor	2.83	− 2.0
N 22	OE2 GLU 104 (B)	H-donor	2.65	− 3.9
N 25	CG1 VAL 24 (B)	H-donor	3.44	− 0.7
N 25	O VAL 25 (B)	H-donor	2.82	− 5.1
N 25	OE1 GLU 104 (B)	H-donor	2.61	− 7.2
N 25	OE2 GLU 104 (B)	H-donor	2.69	− 5.0
C 28	CD1 LEU 162 (B)	H-donor	4.14	− 0.5
C 28	O HOH 310 (B)	H-donor	3.30	− 0.6
C 1	CD1 LEU 159 (B)	H-acceptor	3.52	− 0.7
C 3	CD2 LEU 129 (B)	H-acceptor	3.68	− 0.6
N 7	CE2 PHE 26 (B)	H-acceptor	3.71	− 0.5
N 13	CG1 VAL 24 (B)	H-acceptor	3.42	− 0.9
N 25	CG1 VAL 24 (B)	H-acceptor	3.44	− 0.7
C 28	CD1 LEU 162 (B)	H-acceptor	4.14	− 0.5
N 13	OE2 GLU 104 (B)	Ionic	3.29	− 2.8
N 16	OE2 GLU 104 (B)	Ionic	3.82	− 0.9
N 22	OE1 GLU 104 (B)	Ionic	2.60	− 7.8
N 22	OE2 GLU 104 (B)	Ionic	2.65	− 7.3
N 22	O1P PRP 201 (B)	Ionic	3.68	− 1.3
N 25	OE1 GLU 104 (B)	Ionic	2.61	− 7.7
N 25	OE2 GLU 104 (B)	Ionic	2.69	− 6.9
N 16	6-ring TYR 105 (B)	Cation-pi	3.84	− 0.9

Intriguingly, the kinetin mediated protection from genotoxicity (Fig. 1) may involve the action of APRT. Recently it was demonstrated that DNA is oxidized via the Fenton reaction by ROS; this culminates in the excision of kinetin-riboside by the DNA damage-repair machinery^18^. The enzyme APRT salvages the excision product kinetin to kinetin triphosphate (KTP). KTP, which is an ATP analogue and is used as a phosphate donor by Casine Kinase 2 (CK2) to modify DNA-repair proteins in favor of cell survival and DNA-repair mechanisms^18,29^. Thus, the exogenous administration (or endogenous production) of kinetin potentiates cellular DNA-repair mechanism. Our structural analysis thus pinpoints vital residues that may affect the binding of kinetin to APRT in rescuing cells from genotoxic conditions. In this regard, the outcome of our molecular dynamic simulations can be exploited for functional and genetic approaches (generation of mutants for the mentioned sites) to further explore the binding dynamics of kinetin to the enzyme APRT.

### A2A-R examined as potential cell surface binding site for kinetin and its comparison with the kinetin binding CHASE-domain of histidine kinases in plants

Looking at the various biological activities^18^ CKs perform, such as anti-senescence, anti-inflammation, anti-oxidation and anti-cancer^30^, the metabolic enzymes such as APRT might not be the only proteins that kinetin interacts within the mammalian cells. Rather there is a vast array of non-enzymatic proteins (receptors, transcription factors and regulatory factors) expected to interact with kinetin once administrated to/or produced by the cells. We look now in structural detail at the best suggested targets, conveying the observed kinetin effects and comparing plant cytokinin sensing receptors proteins to analogous mammalian counterparts. One of these putative targets is the adenosine A2A-receptor, where the binding of zeatin-riboside has been shown to prevent the serum-induced cellular apoptosis by acting on this receptor^14^. Most of the CKs are adenine derived regulatory molecules that bind to the CHASE-domain of histidine kinases (AHKs) in plants and microbial cells^8,31,32^. Structurally, adenosine is an adenine with added ribose sugar that binds to A2A-R, and so does the naturally occurring CK zeatin-riboside^14^. Whereas the role of A2A-R is well established in cellular apoptosis, we anticipate that kinetin prevents cytotoxicity and apoptosis in various cell lines (Fig. 1) by acting upon the A2A-R receptor in mammalian cells.

To get insights into the binding dynamics of kinetin to mammalian A2A-R, we compared structural and sequence homology between the binding socket of adenosine to A2A-R and that of CHASE-domain in AHK4 to which kinetin binds. We compared the sequence of the *A. thaliana* AHK4-CHASE-domain (PDB ID: 3T4S^33^) with human A2A-R protein and did not find any significant homology (Supplementary Fig. S1). We performed structural alignment between these two proteins in MOE and found no significant overlap between their PDB derived crystal structures. Furthermore, we visualized the crystal structure of *A. thaliana* AHK4 complex with kinetin and compared it with crystal structure of human A2A-R complex with adenosine. We located the interacting residues of both these complexes; AHK4 A chain residues Asp262 and Leu 284 interact with kinetin (Fig. 3, lower panel). Likewise, A2A-R residues Glu169, Asn253, Ser277 and His278 interact with adenosine (Fig. 3, Table 2). Apparently, there are no common residues between AHK4 and A2A-R in their respective binding pockets for these two-adenine derived small molecules. This is well in line with the long-standing knowledge that plants are unique among the higher eukaryotes that sense cytokinin with a TCS pathway and that cytokinin/kinetin perceiving CHASE-domains^33^ are not present in mammalian cells.

**Table 2:** Ligand interactions report for the binding of adenosine and kinetin to A2A-R receptor.

Ligand	Receptor	Interaction	Distance	E (kcal/mol)
**Adenosine interactions to A2A-R**				
O5′ 1	O HOH 2017 (A)	H-donor	2.73	− 1.0
O3′ 6	OG SER 277 (A)	H-donor	2.79	− 1.8
O2′ 8	O HOH 2016 (A)	H-donor	2.74	− 1.4
N6 15	OE2 GLU 169 (A)	H-donor	3.06	− 4.3
N6 15	OD1 ASN 253 (A)	H-donor	3.11	− 3.3
O5′ 1	O HOH 2017 (A)	H-acceptor	2.73	− 1.0
O3′ 6	OG SER 277 (A)	H-acceptor	2.79	− 1.7
O2′ 8	O HOH 2016 (A)	H-acceptor	2.74	− 1.0
N7 12	ND2 ASN 253 (A)	H-acceptor	3.60	− 2.2
N1 16	O HOH 2018 (A)	H-acceptor	2.72	− 1.3
N3 18	O HOH 2016 (A)	H-acceptor	3.04	− 2.4
5-ring	6-ring PHE 168 (A)	pi-pi	3.61	− 0.0
6-ring	6-ring PHE 168 (A)	pi-pi	3.80	− 0.0
**Kinetin interactions to A2A-R**				
CAM 1	CG2 THR 88 (A)	H-donor	3.96	− 0.5
CAO 3	CG2 THR 88 (A)	H-donor	3.70	− 0.7
CAN 5	CG2 VAL 84 (A)	H-donor	3.56	− 0.8
CAP 9	CD2 LEU 249 (A)	H-donor	4.03	− 0.6
C2 15	CG LEU 249 (A)	H-donor	3.50	− 0.5
N3 17	OD1 ASN 253 (A)	H-donor	2.83	− 3.6
N7 21	CD1 ILE 274 (A)	H-donor	3.52	− 0.5
N9 24	CG GLU 169 (A)	H-donor	3.48	− 0.7
N9 24	OE2 GLU 169 (A)	H-donor	2.80	− 4.4
N9 24	CE MET 270 (A)	H-donor	3.33	− 0.9
CAM 1	CG2 THR 88 (A)	H-acceptor	3.96	− 0.5
CAO 3	CG2 THR 88 (A)	H-acceptor	3.70	− 0.7
CAN 5	CG2 VAL 84 (A)	H-acceptor	3.56	− 0.8
CAP 9	CD2 LEU 249 (A)	H-acceptor	4.03	− 0.6
N6 12	O HOH 2016 (A)	H-acceptor	3.02	− 1.3
C2 15	CG LEU 249 (A)	H-acceptor	3.50	− 0.5
N7 21	CD1 ILE 274 (A)	H-acceptor	3.52	− 0.5
N7 21	O HOH 2016 (A)	H-acceptor	2.86	− 3.0
N9 24	CG GLU 169 (A)	H-acceptor	3.48	− 0.7
N9 24	CE MET 270 (A)	H-acceptor	3.33	− 0.9
5-ring	NE2 HIS 278 (A)	pi-H	3.36	− 1.4

We then docked kinetin to A2A-R and found that kinetin does not bind to A2A-R in an adenosine-like pattern using a repertoire of molecular interactions (Fig. 3, Table 2). More intriguingly, we found that there is another active site like pocket where kinetin binds to A2A-R and that kinetin binding dynamics to A2A-R are different from the binding of adenosine (Table 2). Future studies focusing on the kinetin binding residues of A2A-R will help elucidate the molecular basis of kinetin actions in animal cells. The alternative binding sites on A2A-R nominate kinetin as potential agonist for dealing with oxidative stress arising from various pathophysiological conditions.

### Future perspective and work on kinetin-binding proteins (KBPs) and kinetin mediated metabolic and transcriptional changes in mammalian cells

To further understand the molecular basis of protection kinetin confers to mammalian cells against oxidative stress, we discuss few methodologies that will shed light on the detailed understanding of kinetin functions in mammalian cells at the molecular level. Desired cells will be treated with kinetin, kinetin-riboside or H_2_O_2_ (to envision the effect of oxidative stress: Fig. 1) prior to RNA isolation and the generation of transcriptome datasets. Likewise, metabolomes datasets can be generated by using GCMS after treating cells with kinetin. Both transcriptomes and metabolomics datasets can be combined to assess a genome scale metabolic network model in order to infer about the metabolic capacity endowed by the kinetin treatment to the cells. Likewise, kinetin-immobilized resins or beads and columns can be prepared to scrutinize KBPs in the cellular proteomes. Protein extract from the target cells can be loaded onto a kinetin-linked resin. The loaded column will be washed to remove non-specifically bound proteins. Column-retained proteins will be eluted with loading buffer containing high concentration (mM) kinetin and will be analyzed. Eluted proteins will then be subjected to mass spectrometry. Selected proteins can be characterized and their affinity kinetics to kinetin can be assessed. These and alike approaches will further underscore the molecular mechanisms of the protection kinetin confers to mammalian cells against oxidative stress.

In conclusion, our analysis underscores the critical kinetin concentrations that protect cells from cytotoxicity under normal and pathophysiological conditions. The endogenous production of kinetin under stressed conditions or its exogenous application can be metabolized by animal cells and the enzyme APRT plays an important role in the metabolism of kinetin. We identified important residues in the catalytic cleft of APRT that facilitate the binding of kinetin to this evolutionary conserved enzyme across different life forms. Our computational and structural analysis provides evidence of the binding dynamics of kinetin and the human A2A-R receptor. Future concerted efforts in finding kinetin and CK drug targets in the human proteome will be instrumental in harnessing the applied benefits of kinetin as an alley to a new generation of pharmaceuticals.

## Supplementary information

Supplementary Figure S1.

## Data Availability

All data are contained in the submitted files and figures.
