# Supplementary material for: Integrated structural and functional analysis of the protective effects of kinetin against oxidative stress in mammalian cellular systems
Source: Sci Rep. 2020 Aug 7;10:13330. doi: 10.1038/s41598-020-70253-1 (PMC7414151; doi:10.1038/s41598-020-70253-1)
Supplement: Supplementary file 1 — Supplementary Figure S1. [file 41598_2020_70253_MOESM1_ESM.pdf]

Integrated structural and functional analysis of the protective effects of kinetin against oxidative stress in mammalian cellular systems

Muhammad Naseem[1 2 *$], Eman M. Othman[2 3*], Moustafa Fathy[3,4], Jibran Iqbal[1], Fares M. Howari[1], Fatima A. AlRemeithi[1], Geema Kodandaraman[5], Helga Stopper[5], Elena Bencurova[2], Dimitrios Vlachakis[6] and Thomas Dandekar [2 $]

1. College of Natural and Health Sciences, Department of Life and Environmental Sciences, Zayed University, Abu Dhabi, UAE
2. Department of Bioinformatics, Biocenter, University of Würzburg, Am Hubland, Wuerzburg, Germany
3. Department of biochemistry, Faculty of Pharmacy, University of Minia, Minia, Egypt,
4. Department of Regenerative Medicine, Graduate School of Medicine and Pharmaceutical Sciences, University of Toyama, Toyama, Japan
5. Institute of Pharmacology and Toxicology, University of Würzburg, Würzburg, Germany
6. Genetics Laboratory, Department of Biotechnology, Agricultural University of Athens, 75 Iera Odos str, 11855, Athens, Greece
[*]These authors have equally contributed to this work

[$]correspondence should be addressed to: muhammad.naseem@zu.ac.ae and dandekar@biozentrum.uni-wuerzburg.de

Supplementary Material: Figure S1

The **Figure S1B** was made by the authors using Molecular Operating Environment Suite (MOE by CCG) as described in Materials and Methods. More details are found in the following reference

Vlachakis D, Kossida S. Molecular modeling and pharmacophore elucidation study of the Classical Swine Fever virus helicase as a promising pharmacological target. PeerJ. 2013 Jun 11;1:e85.

Details on MOE: Molecular Operating Environment (MOE) (2011) Molecular Operating Environment (MOE 2011.10). Montreal, Quebec, Canada: Chemical Computing Group, Inc. Available: http://www.chemcomp.com

A

```
3T4S:  ---MDDANKIRRE----------EVLV-------------------SM--CDQRARMLQD   26
2YDO:  MPIMGSSVYITVELAIAVLAILGNVLVCWAVWLNSNLQNVTNYFVVSAAAADILVGVLAI   60
          *..:  *  *          :***                   *   .*  . :*

3T4S:  QFSVSVNHVHA----LAILVSTFHYHKNPS------AIDQETFAEYTARTAFERPLLSGV   76
2YDO:  PFAIAISTGFCAACHGCLFIACFVLVLTASSIFSLLAIAIDRYIA------IRIPL----   110
        *::::.  ..     .:::: *    . *      **  : :        :. **

3T4S:  AYAEKVVNFEREMFERQHNWVIKTMDRGEPSPVRDEYAPVIFSQDSVSYLESLDMMSGEE   136
2YDO:  RYNGLVTG-TRAKGIIAICWVLSFA-------------------------IGLTPMLGWN   144
        *   *..  *        **:.                            .*  * * :

3T4S:  ----DRENILRARETGKAVLTSPFR------------LLETHHLGVVLTFPVYKSS----   176
2YDO:  NCGQPKEGKAHSQGCGEGQVACLFEDVVPMNYMVYFNFFACVLVPLLLMLGVYLRIFLAA   204
            :*.  :::  *:. ::. *.            ::    : ::* : **

3T4S:  ------LPENPTVEERIA----------ATAGYLGGAFDVESLVENLLGQL---A---GN   214
2YDO:  RRQLKQMESQPLPGERARSTLQKEVHAAKSLAIIVGLFALCWLPLHIINCFTFFCPDCSH   264
             : .:*  **             : . : * * :  *  :::. :   .   .:

3T4S:  QAIVV----HVYDITN-ASDPLVMYGNQDEEA---DRSLSHESKLDFGDPFRKHKMICRY   266
2YDO:  APLWLMYLAIVLSHTNSVVNPF-IYAYRIREFRQTFRKIIRSHVLRQQEPFKAAAAENLY   323
         : :     * . ** . :*: :*. : .*     *.: :.  *   :**:       *

3T4S:  HQKA   270
2YDO:  FQ--   325
       .*
```

B

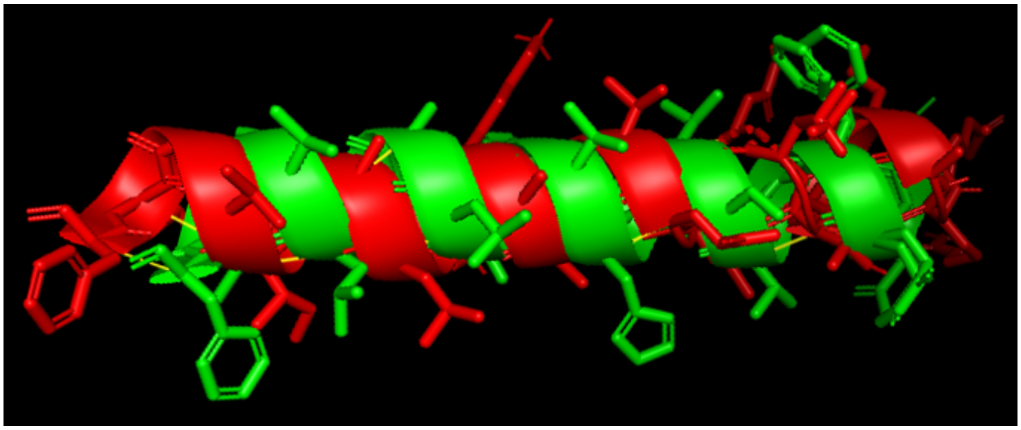

Supplementary Figure 1: Homology between Arabidopsis AHK4 and Human A2A-R receptors

(A) Local pairwise sequence alignment between AHK4 and A2A-R proteins. The CHASE-domain (cyclases/histidine kinases associated sensory extracellular domain) falls between the two arrows on AHK4 sequence.

(B) Structure comparison of *A.thaliana* AHK4 A chain and human A2A-R structures (Green: AHK4, Red: A2A-R). Structures are in their sequence details quite different, share in the part shown a helical structure; done using MOE software and the available PDB coordinates).
